# Alkaloids from the Tribe Bocconieae (Papaveraceae): A Chemical and Biological Review

**DOI:** 10.3390/molecules190913042

**Published:** 2014-08-25

**Authors:** Xuelong Yu, Xiaoli Gao, Zhixiang Zhu, Yuan Cao, Qian Zhang, Pengfei Tu, Xingyun Chai

**Affiliations:** 1 Modern Research Center for Traditional Chinese Medicine, Beijing University of Chinese Medicine, Beijing 100029, China; 2 School of Chinese Materia Medica, Beijing University of Chinese Medicine, Beijing 100102, China

**Keywords:** Papaveraceae, *Macleaya*, *Bocconia*, alkaloids, bioactivity, review

## Abstract

The Bocconieae tribe, consisting of only the genera *Macleaya* and *Bocconia*, possesses significant economic and medicinal value and plays an important role in health management for people in developing countries. During the past decades, research on metabolites and relative pharmacology, including the isolation and identification of a variety of molecules, has shed light on the tribe. Among those molecules, isoquinoline alkaloids, and their antimicrobial, antifungal, and anti-inflammatory activities are especially noteworthy. This paper presents a comprehensive compilation of current research progress, with emphasis on the alkaloids and their distribution, phytochemical and pharmacological investigation, toxicity and side effects, related chemotaxonomy and future use prospects, and hopefully provides a valuable reference as an effort to promote further exploration and application of this tribe.

## 1. Introduction

The Papaveraceous plants are very well-known for their extensive economic use as well as medicinal ones, which are directly associated with their rich production of alkaloids with novel structures and significant bioactivities. A variety of alkaloids, such as morphine, codeine, protopine, isocorydine, and tetrahydropalmatine, were discovered in this family, and they have irreplaceable therapeutic value in the treatment of many diseases.

The Bocconieae is a small tribe consisting of only two closely related genera, *Macleaya* and *Bocconia*, which share similarities in morphology and chemical components. The genus *Macleaya* contains two slightly toxic species, *M. cordata* (Willd.) R. Br. and *M. microcarpa* (Maxim.) Fedde, distributed in East Asia and widely found in South and Northwest China [1]. The genus *Bocconia*, on the other hand, includes *ca.* nine species, distributed mainly in tropical areas of Mexico, Central and Southern America [2].

The plants of the tribe Bocconieae play an important role as sources of traditional medicines for people in developing countries. For example, *B. frutescens* is used in Mexico to treat skin ulcers, dermatitis, and some respiratory tract infections, as well as tuberculosis [3]. *B. arborea* is employed for the treatment of diverse infectious diseases and is variously known in Mexico as llora sangre (weeping blood), cocoxíhuitl, ahuacachilli, mano de león (lion’s hand), palo del diablo (devil’s stick), palo amarillo (yellow stick). Native residents also use *B. arborea* as a purgative, vermifuge, antitumor, and anti-inflammatory agent to heal wounds and dissolve warts, or as a carminative agent to take advantage of its cathartic and analgesic activities [2,4]. *M. cordata* has been widely used as folk medicine in China, North America, and Europe, where it has been applied to cure cervical cancer and thyroid cancer, according to clinical records [5].

Modern chemistry and pharmacology research has revealed that alkaloids are the characteristic ingredients, overwhelming in quantity, and account for the major bioactivities of most Papaveraceous plants [6]. Therefore, this paper compiles complete data of alkaloids from the plants of the tribe Bocconieae, focusing on their distribution, isolation, structural features, and pharmacological activities, and hopefully may provide a useful reference for further studies on this tribe.

## 2. Chemical Constituents

**Figure 1:**
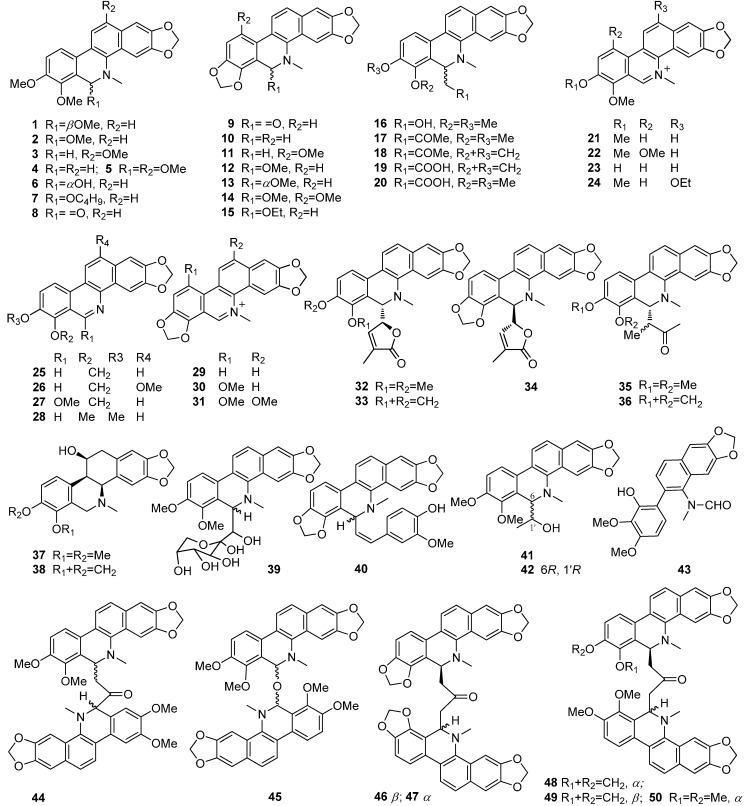
Structures of compounds **1**–**50** from the tribe Bocconieae.

**Figure 2:**
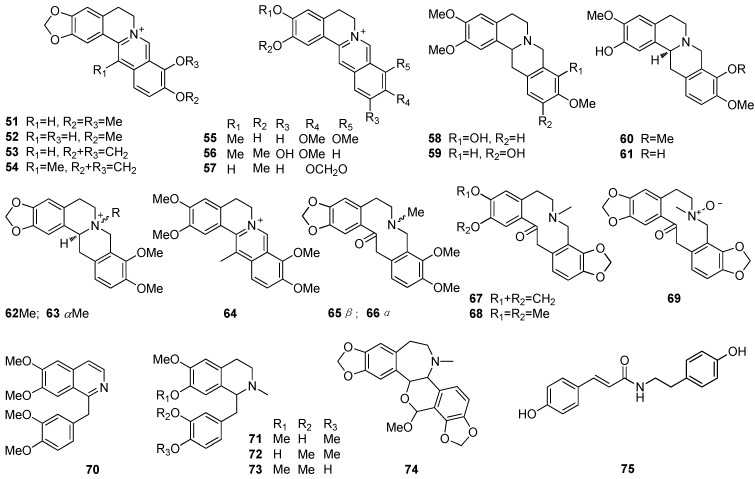
Structures of compounds **51**–**75** from Bocconieae.

A total of 75 alkaloids have been described from Bocconieae species so far, including the predominant benzophenanthridines (BPAs) **1**–**50**, protoberberines **51**–**64**, protopines **65**–**69** and other types **70**–**75**. Their structures are displayed in Figure 1 and Figure 2, and their names, corresponding source plants, parts, and references are listed in Table 1 and Table 2.

The BPA type of alkaloids, derived from protoberberines via *N*-*C*_6_ bond cleavage and the formation of *C*_6_-*C*_13_ bonds is one of the characteristic and chemotaxonomic components in the plants of the family Papaveraceae [7,8]. The BPAs in the plants of the tribe Bocconieae cover dihydrobenzophen-anthridines (**1**–**20**, **32**–**36**, **39**–**42**), quaternary BPAs (QBPAs, **21**–**24**, **29**–**31**), *N*-demethylated BPAs **25**–**28**, hexahydrobenzophenanthridines **37**–**38**, dimeric BPAs **44**–**50**, and the *seco*-BPA **43**. Among them, some (compounds **4**, **9**, **10**, **17**, **18**, **21**, **29**, **48**, **49**, **66**, and **67**) are widespread, especially **21**, **29**, and **65**–**67**, and some (compounds **4**, **8**–**10**, **16**, and **30**) are less abundant, while **51**, **53** and **54** are scarce in this tribe. Dimers **44**–**50** from *B. arborea* and *M. microcarpa* are considered artifacts probably derived from reactions occuring during the extraction and isolation, although the possibility their natural existence cannot be completely excluded, as similar dimers were also described in plants of the genus *Dactylicapnos* in the same family [9]. Compound **7** should not be a natural product, as it is obviously derived from the use of *n*-butanol during its extraction. Furthermore, compounds **56**, **58**, **59**, and **70**–**73** are obtained in cell cultures of *M. cordata.*

In addition to the major BPA alkaloids, other types of constituents, including phenolic acids, essential oil (2-methoxy-4-vinylphenol) [10,11], polysaccharides, flavonoids, steroids [12] from *M. cordata*, and a triterpene (3*α*-hydroxyolean-12-en-30-oic acid) from *B. arborea* [13] were also reported.

Besides distribution and structural features, determination of stereochemistry of the alkaloids is another important issue, not only for the structural elucidation, but also for the clarification of reaction mechanism(s), especially for those alkaloids with complex skeletons and more than one chiral center. The relative configurations of compounds **17** and **32**–**34** were solved by X-ray single-crystal diffraction and analysis of circular dichroism data [14,15]. However, the absolute configurations of many of these alkaloids remaining unresolved, and in need of further research efforts.

**Table 1:** Alkaloids isolated from Bocconieae.

	Name		Type			Plant		Part	Ref.
**1**	11-*O*-Methyldihydrochelerythrine		I_A_			*B. arborea*		—	[16]
**2**	Angoline ((±)-6-methoxydihydro-chelerythrine)		I_A_			*M. cordata*		Stems	[17]
						*B. arborea*		Leaves, stems	[2]
						*M. microcarpa*		Aerial parts, roots	[14,18,19]
**3**	12-Methoxydihydrochelerythrine		I_A_			*B. integrifolia*		Leaves	[20]
**4**	Dihydrochelerythrine		I_A_			*B. integrifolia*		Leaves	[20]
						*B. arborea*		Aerial parts	[4]
						*B. frutescens*		Leaves	[3]
						*M. microcarpa*		Roots, leaves, whole plant	[14,21,22]
						*M. cordata*		Fruits	[23]
						*B. pearcei*		Fruits	[24]
**5**		6, 12-Dimethoxydihydrocheleritrine	I_A_			*B. arborea*		—	[25]
**6**		8-Hydroxydihydrochelerythrine	I_A_			*M. cordata.*		Seeds	[26]
**7**		6-Butoxydihydrochelerythrine	I_A_			*M. microcarpa*		Roots	[14]
**8**		Oxychelerythrine	I_A_			*B. pearcei*		Fruits	[24]
**9**		Oxysanguinarine	I_A_			*B. latisepala*		Leaves, roots, seeds	[27]
						*B. arborea*		—	[16]
						*B. pearcei*		Fruits	[24]
						*M. cordata*		—	[10]
**10**		Dihydrosanguinarine	I_A_			*M. cordata*		Fruits	[23]
						*B. integrifolia*		Leaves	[20]
						*B. arborea*		Aerial parts	[4,16]
						*M. microcarpa*		Roots, leaves, whole plant	[14,21,22]
						*B. Pearcei*		Fruits	[24]
**11**		Dihydrochelirubine	I_A_			*B. integrifolia*		Leaves	[20]
						*B. pearcei*		Fruits	[24]
**12**		6-Methoxydihydrosanguinarine	I_A_			*M. cordata*		Fruits	[10]
						*M. microcarpa*		Roots	[14]
**13**		8-Methoxydihydrosanguinarine	I_A_			*M. cordata*		Seeds	[26]
**14**		6-Methoxydihydrochelirubine	I_A_			*B. arborea*		—	[25]
**15**		6-Ethoxysanguinarine	I_A_			*M. cordata*		—	[10]
						*M. microcarpa*		Whole plant	[21]
**16**		Bocconoline	I_A_			*B. cordata*		—	[28]
						*M. cordata*		—	[10]
**17**		(±)-6-Acetonyldihydrochelerythrine	I_A_			*B. arborea*		Barks, aerial parts	[2,15]
						*B. frutescens*		Leaves	[3]
						*M. cordata*		Fruits	[10,23]
**18**		(±)-6-Acetonyldihydrosanguinarine	I_A_			*B. arborea*		Aerial parts	[2]
						*B. frutescens*		Leaves	[3]
						*M. cordata*		Fruits	[10,23]
						*M. microcarpa*		Whole plant	[21]
**19**		Spallidamine(6-Carboxymethyldihydrosanguinarie)	I_A_			*M. microcarpa*		Roots	[14]
						*M. cordata*		Whole plant	[12]
**20**		6-Carboxymethyldihydrochelerythrine	I_A_			*M. cordata*		Whole plant	[12]
**21**		Chelerythrine	I_B_			*B. latisepala*		Barks, stems, seeds	[27]
						*M. cordata*		—	[29]
						*B. frutescens*		Roots, stalks and leaves	[30]
						*M. microcarpa*		—	[31]
**22**		Chelilutine	I_B_			*M. cordata*		Roots	[10]
						*M. microcarpa*		—	[31]
**23**		8-*O*-Demethylchelerythrine	I_B_			*M. cordata*		—	[29]
**24**		6-Ethoxychelerythrine	I_B_			*M. cordata*		—	[10]
**25**		Norsanguinarine	I_C_			*M. cordata*		Fruits	[10]
**26**		12-Methoxynorchelerythrine	I_C_			*B. pearcei*		Fruits	[24]
**27**		Pancorine	I_C_			*M. microcarpa*		Roots	[14]
**28**		Norchelerythrine	I_C_			*M. cordata*		Whole plant	[12]
**29**		Sanguinarine	I_B_			*B. latisepala*		Barks, stems	[27]
						*B. cordata*		Leaves	[32]
						*B. frutescens*		Roots, stalks and leaves	[30]
						*M. cordata.*		Fruits	[29,33]
						*M. microcarpa*		Aerial parts	[18,19,34]
**30**		Bocconine (chelirubine)	I_B_			*B. cordata*		—	[35]
						*M. microcarpa*		—	[31]
**31**		Macarpine	I_B_			*M. cordata*		Callus tissues	[10,36]
						*M. microcarpa*		—	[31]
**32**		Maclekarpine A	I_A_			*M. microcarpa*		Roots	[14]
**33**		Maclekarpine B	I_A_			*M. microcarpa*		Roots	[14]
**34**		Maclekarpine C	I_A_			*M. microcarpa*		Roots	[14]
**35**		6 *α*-Isobutanonyldihydrochelerythrine	I_A_			*M. cordata*		Fruits	[23]
**36**		6 *α*-Isobutanonyldihydrosanguinarine	I_A_			*M. cordata*		Fruits	[23]
**37**		Homochelidonine	I_D_			*M. cordata*		—	[10]
**38**		Chelidonine	I_D_			*B. frutescens*		Roots	[37]
**39**		Maclekarpine D	I_A_			*M. microcarpa*		Roots	[14]
**40**		Maclekarpine E	I_A_			*M. microcarpa*		Roots	[14]
**41**		6-(1'-hydroxyethyl)-dihydrochelerythrine	I_A_			*M. microcarpa*		Roots	[14]
**42**		*R*-6-((*R*)-1-Hydroxyethyl)-dihydrochelerythrie	I_A_			*M. cordata*		Whole plant	[12]
**43**		Arnottianamide	I_F_			*M. microcarpa*		Roots	[14]
**44**		Chelerythridimerine	I_E_			*B. arborea*		Barks	[38]
**45**		Bis[6-(5, 6-dihydrochelerythrinyl)]ether	I_E_			*M. microcarpa*		Roots	[14]
**46**		(±)-Sanguidimerine	I_E_			*B. arborea*		Aerial parts	[2]
						*M. cordata*		Leaves	[10]
**47**		Chelidimerine	I_E_			*B. arborea*		Aerial parts	[2]
						*M. cordata*		Leaves	[10]
**48**		(±)-Bocconarborine A	I_E_			*B. arborea*		Aerial parts	[2]
						*M. cordata*		Leaves	[10]
						*M. microcarpa*		Whole plant	[21]
**49**		(±)-Bocconarborine B	I_E_			*B. arborea*		Aerial parts	[2]
						*M. cordata*		Leaves	[10]
**50**		1, 3-Bis(1l-hydrochelerythriny1)acetone	I_E_			*B. arborea*		—	[16]

I: benzophenanthridine (BPA); I_A_: dihydrobenzophenanthridine; I_B_: QBPA; I_C_: *N*-demethylation BPA; I_D_: hexahydrobenzophenanthridine; I_E_: dimeric BPA; I_F_: *seco*-BPA.

**Table 2:** Alkaloids isolated from Bocconieae.

	Name	Type	Plant	Part	Ref.
51	Berberine	II	*B. frutescens* *M. cordata* *M. microcarpa*	Roots, stalks, leaves — Roots	[30] [10] [31]
52	Berberrabine	II	*M. microcarpa*	Whole plant	[21]
53	Coptisine	II	*B. frutescens* *M. microcarpa*	Roots, stalks and leaves Roots	[30] [31]
54	Corysamine	II	*B. frutescens*	—	[37]
55	Columbamine	II	*B. frutescens*	Roots, stalks, leaves	[30]
56	Dehydrocorytenchine	II	*M. cordata*	Cultured cells	[39]
57	Dehydrocheilanthifoline	II	*M. cordata*	—	[40]
58	Tetrahydropalmatrubine	II	*M. cordata*	Cultured cells	[39]
59	Corytenchine	II	*M. cordata*	Cultured cells	[39]
60	(–)-Isocorypalmine	II	*B. frutescens*	Leaves, roots	[30,37,41]
61	(–)-Scoulerine	II	*B. frutescens*	Roots, leaves	[30,37]
62	(–)-*cis-N-*Methylcanadinium	II	*B. frutescens*	Roots	[37]
63	(–)-*α*-Canadine	II	*B. frutescens*	Roots, stalks, leaves	[30]
64	Dehydrocicanthifoline	II	*M. cordata*	—	[40]
65	*β*-Allocryptopine	III	*M. microcarpa*	Aerial parts, whole plant	[18,19,21]
66	*α*-Allocryptopine	III	*B. cordata* *M. cordata* *B. latisepala* *M. microcarpa*	Leaves Fruits Roots Whole plant	[32] [10] [27] [21]
67	Protopine	III	*B. latisepala* *B.cordata* *B. frutescens* *M. cordata* *M. microcarpa*	Leaves, roots Leaves Roots, stalks, leaves Cultured cells Aerial parts, whole plant	[27] [32] [30,41] [39] [19,21]
68	Cryptopine	III	*M. cordata* *M. microcarpa*	Fruits Aerial parts	[10] [18]
69	Protopine *N*-oxide	III	*B. cordata*	Whole plant	[10,42]
70	Papaverine	IV	*M. cordata*	Cultured cells	[39]
71	Laudanine	IV	*M. cordata*	Cultured cells	[43]
72	Codamine	IV	*M. cordata*	Cultured cells	[43]
73	Pseudocodamine	IV	*M. cordata*	Cultured cells	[43]
74	Rhoeadine	IV	*B. frutescens*	Leaves, stalks	[30,41]
75	*N-p*-Coumaroyltyramine	IV	*M. microcarpa*	Roots	[14]

II: protoberberine; III: protopine; IV: other types.

## 3. Biological Activities

Crude extracts, essential oils and other individual compounds from the plants of the tribe Bocconieae display insecticidal, cough treatment, antitumor and antifungal activities. Among all investigations, *M. cordata* is the most addressed species.

### 3.1. Cytotoxicity against Tumor Cells

The *in vitro* anticancer properties of crude extract of *M. cordata* were assessed against MRC5 fetal lung fibroblasts and A549 adenocarcinomic epithelial cells. Viability of the treated MRC5 cells was reduced in a concentration-dependent manner, demonstrating that the normal lung cells are sensitive to the extract. Surprisingly, the A549 viability was slightly increased in response to extract exposure at a low concentration, whereas the viability was reduced accordingly at higher concentrations [44]. Fractions from the ethanol extract of the roots of *M. microcarpa* exhibited cytotoxicity against the Bel-7402, BGC-823, HCT-8, A2780 and A549 cell-lines, with IC_50_ values in the 1.1–23.8 μg/mL range [14].

The total alkaloids of *M. cordata* showed *in vivo* inhibitory activity against transplanted tumors in mice in a dose-dependent manner [45], and further *in vitro* assessment of total alkaloids of *M. cordata* showed significantly inhibitory activity against the proliferation of human Hep3B cells and murine H22 cells, with IC_50_ values of 2.0–3.0 μg/mL. The total alkaloids of *M. cordata* also inhibited the development of tumors and prolonged the survival of S180 tumor-bearing mice [46].

It was reported that chelerythrine (CHE, **21**) inhibited the proliferation of BGC-823 human gastric cancer cells in a time- and dose-dependent manner, accompanied with S phase arrest. It also induced apoptosis by a mechanism involving a reduction in the mitochondrial membrane potential, the release of cytochrome c, activation of caspase 3 and cleavage of poly-ADP-ribose polymerase (PARP). In addition, CHE-induced apoptosis was accompanied by down-regulation of Bcl-xl and Bcl-2 proteins without change in the levels of Bax proteins [47]. Compounds **7**, **12** and **34** displayed marked cytotoxicities against the Bel-7402, BGC-823, HCT-8, A2780 and A549 cell-lines, with IC_50_ values of 0.5–3.4 μM, equal to the positive control camptothecin (0.28 to 3.15 μM) [14]. Adhami *et al* demonstrated that sanguinarine (SAN, **29**) caused cell cycle blockade and apoptosis of human prostate carcinoma cells via modulation of cyclin kinase inhibitor-cyclin-cyclin-dependent kinase machinery [48]. Furthermore, SAN induced apoptosis of human pancreatic carcinoma AsPC-1 and BxPC-3 cells via modulations in Bcl-2 family proteins [49], and induced apoptosis in A549 human lung cancer cells primarily via cellular glutathione depletion [50].

Besides, protopine (PRO, **67**), cryptopine (CRY, **68**) and allocryptopine (ALL, **65**/**66**) potently inhibited human cytochrome P450 (CYP) 2D6, with IC_50_ values lower than 1 μM. PRO and CRY moderately inhibited CYP2C19 with IC_50_ values of 1–10 μM [51].

### 3.2. Insecticidal Activities

Ethanolic extract of the seeds of *M. cordata* showed a significant insecticidal effect against the growth of the cotton aphid *Aphis gossypii* Glover [26]. Crude extracts and fractions from the leaves of *M. cordata* were investigated *in vitro* against the fish parasite *Ichthyophthirius multifiliis* by a bioactivity-guided isolation method, and caresults showed that chloroform extract exhibited promising activity with 100% antiparasitic efficacy at the concentration of 70.0 mg/L after 4 h of exposure [52]. The chloroform extract of the leaves of *M. microcarpa* also showed a promising antiparasitic activity against *I. multifiliis* [22].

Compounds **4** and **10** showed potent activity against *I. multifiliis* with EC_50_ values of 9.43 mg/L and 5.18 mg/L, respectively, after 48 h of exposure [22]. SAN (**29**) exhibited a remarkable inhibitory effect against *I. multifiliis* at a concentration of 0.7 mg/L, with an LC_50_ value of 0.437 mg/L after 4 h of exposure. *In vivo* antiparasitic efficacy tests showed that the number of *I. multifiliis* on the gills in the treatment group (in 0.9 mg/L SAN) was reduced by 96.8% in comparison to the untreated at 25 °C for 48 h. There were no deaths in the treatment group during the trial compared with a 40% death rate of the untreated fish [52]. Compound **10** showed significant inhibitory activity against *Leishmania* with an IC_50_ value of 0.014 μg/mL, followed by compounds **4** and **11** with the same IC_50_ value of 0.166 μg/mL [24]. Compounds **6** and **13** demonstrated an effect in decreasing the survival rate of the cotton aphid by 76.1% ± 7.9% and 73.6% ± 14.6% at 100 ppm, respectively [26]. The bisulfates of CHE, SAN and total alkaloids from the fruits of *M. cordata* have molluscicidal activities against the snail *Oncomlania hupensis*, the intermediate host of schistosomiasis, in both time-and concentration-dependent manners, with LC_50_ values at 72 h of 2.05, 0.19, and 0.40 mg/L, respectively [53]. 

Wang *et al.* reported that the extract of aerial parts of *M. microcarpa* and isolates (**2**, **29**, **65**, **67**, and **68**), especially SAN (**29**), might be useful for the treatment of *Dactylogyrus intermedius* infections. Another *in vivo* anthelmintic assay backed the theory by reporting evident inhibitory effects with EC_50_ values of 0.37–8.13 mg/L [19].

### 3.3. Antimicrobial Activity

The plants of Bocconieae species, such as *B. arborea* [54] and *B. frutescens* [3], exhibited strong antimicrobial activity, therefore they have been applied extensively as traditional medicines for the treatment of diverse infectious diseases.

Methanolic extract of the leaves of *B. arborea* showed a general antimicrobial effect against *Staphylococcus aureus*, *Escherichia coli*, and *Pseudomonas aeruginosa* at 10 mg/mL and below [55]. Both ethanolic extract of leaves and hexane extract of stems of *B. frutescens* showed strong activities against *E. coli* and *S. aureus* [3]. Moreover, methanol and hexane extracts of *B*. *frutescens* leaves displayed *in vitro* antimycobacterial activity against *Mycobacterium tuberculosis* with the same MIC value of 125 μg/mL [56].

The bisulfates of quaternary benzophenanthridines from *M. cordata* showed strong inhibitory effects against *Elsinoe ampelina*, *Colletotrichum gloeosporioides*, *Cercospora viticola*, *Pyricularia oryzae*, *Gibberella zeae*, and *Phytophythora capsici*, with EC_50_ values of 3.35–10.08 μg/mL [57]. Also, extracts from *M. cordata* formulated at 150 mg/L QBPA are used to spray greenhouse roses (*Rosa* sp.) infected by *Sphaerotheca pannosa* var. *rosae* (powdery mildew) at 10-day intervals. One day after application, symptoms of mildew infection show visible reduction by 60%. Subsequent studies demonstrated that a tank of QBPAs provided enhanced control of powdery mildew on roses [58].

Methanolic extract of *B. frutescens* showed moderate inhibitory activity against *Trichomonas vaginalis* with annn IC_50_ value of 30.9 μg/mL [59]. Crude alcoholic extracts of different parts of fresh or dried *B. frutescens* possessed antimalarial activity. Compared to dried raw materials, extract of the fresh plant exhibited a higher inhibitory effect against *Plasmodium berghei*, especially of fresh green fruits (IC_50_ 2.4 μg/mL) [60]. QBPAs from the plants of the genus *Macleaya* are effective in the control of many fungal diseases. Methanolic extract of the leaves of *B. arborea* exhibited anti-yeast activity against *Candida albicans* with MIC value of 2.5 mg/mL [55]*.*

Compounds **4** and **10** displayed significant antimicrobial activities against *S. aureus*, *Streptococcus faecalis*, *Proteus mirabilis*, and *E. coli* with MIC values of 9.3–300 μg/mL [4]. Besides, they also exhibited the highest *in vitro* antifungal activity against *Botrytis cinerea* Pers with inhibitory rates of 98.32% and 95.16% at 50 μg/mL, respectively, for they inhibit spore germination in a concentration-dependent manner. Moreover, they showed potent *in vivo* protective and curative effects on *Erysiphe graminis* and *B. cinerea* [61]. Compounds **2** and **14** both had the same MIC value of 12.5 μg/mL against a sensitive strain of *M. tuberculosis* H37Rv [25]. Compound **2** also exhibited activity against *S. aureus* and *S. faecalis*, with a MIC value around 25 μg/mL [2]. Further, **2**, **4** and **10** exhibited anti-yeast activity against *Candida albicans* with MIC values of 12.0, 18.7 and 18.7 μg/mL, respectively, while nystatin was 5.0 μg/mL [2,4].

CHE (**21**) and SAN (**29**) demonstrated a significant antifungal activity against *Rhizoctonia solani* with IC_50_ values of 0.55 and 0.47 μg/mL, respectively. They are also effective against *Botryosphaeria bernegeriana*, *Botrytis cinerea*, *Fusarium graminearum*, *F. oxysporum* f.sp. *lycopersici*, *F. oxysporum* f.sp. *vasinfectum*, *Magnaportheoryzae*, and *R. solani* [62]. In most of the bacterial strains used, such as *S. aureus* CCM 3953, *S. aureus* CCM 4223, *P. aeruginosa* CCM 3955, two strains of *E. coli* (CCM 4225 and CCM 3954), and *Streptococcus agalactiae*, the antimicrobial activity increased as the concentration increased, which means the pure CHE and SAN exhibited the most potent effect. PRO (**67**) and ALL (**65**/**66**) showed weaker antimicrobial activity than CHE and SAN, and dihydrosanguinarine (**10**) had a mild inhibitory effect with MIC value around 500 μg/mL, whereas **4** was inactive [10].

Besides, angoline (**2**), SAN, ALL, and CRY (**68**) also showed *in vitro* activities against fish pathogenic bacteria *Aeromonas hydrophila*, *A. salmonicida*, *Vibrio anguillarum* and *V. harveyi* with MIC values of 12.5–200 mg/L compared with florfenicol 0.5–2.0 mg/L [18].

### 3.4. Anti-Inflammatory Property

Some of the plants of this tribe have been shown to possess anti-inflammatory properties. The aqueous plant extract of *B. arborea* showed anti-inflammatory activity and is recommended for medical use in the treatment of oral inflammatory processes [38]. Extracts from *M. cordata* are also used in traditional medicine for their anti-inflammatory activity [62,63].

Research on anti-inflammatory activity has been primarily focused on possible interactions with the nuclear factor-κB (NF-κB) pathway which plays an important role in regulating the expression of cyclooxygenase-2 and pro-inflammatory cytokines. In cell models, SAN was found to inhibit NF-κB activation and CHE suppressed inducible expression of cyclooxygenase-2. The anti-inflammatory action of CHE and SAN could also be associated with their ability to inhibit formation of superoxide radical by phagocyte NADPH oxidase. In contrast, the relevant molecular targets of PRO and ALL (**65**/**66**) have not been identified to date [64]. CHE and SAN exhibited local anti-inflammatory effects in the carrageenan-induced pawedema test in rats. SAN but not CHE inhibited the signal transduction pathways critical to the inflammatory response leading to NF-κB activation [63].

### 3.5. Effect on Cardiovascular System

The aqueous extract of *B. frutescens* possessed potent stimulatory effects with an EC_50_ of 18 ± 2.4 μg/mL, which presented maximum contractile response (E_max_ = 80.6 ± 5.6%) when the vascular tone changed [65]. The methanolic and dichloromethane extracts of the roots of *B. frutescens* inhibited [^3^H]-angiotensin II binding (AT II, AT_1_ receptor) by more than 50% [66].

CHE and SAN were significant inhibitors of [^3^H]-AT II binding (hAT_1_ receptor) with IC_50_ values 9.93 and 1.90 μM, respectively. On the other side, the cyclo(-d-Trp-d-Asp-[prolyl-3, 4(n)-[^3^H**]**]Pro-d-Val-Leu) binding ([^3^H]-BQ-123, ET_A_ receptor) was mildly inhibited [37].

SAN interacted with the human AT_1_ receptor in a slow, nearly irreversible and non-competitive manner. The inhibition of [^3^H] (2-ethoxy-1-[(2'-(1*H*-tetrazol-5-yl)biphenyl-4-yl)methyl]-1*H*-benzimid-azoline-7-carboxylic acid) ([^3^H]candesartan) binding by SAN was independent of cell viability, since the alkaloid inhibited both intact Chinese hamster ovary (CHO) cells transfected with human AT_1_ receptor and with their cell membranes (*K*_i_ = 0.14 and 1.10 μM, respectively) at a similar extent radioligand binding [67]. 

### 3.6. Other Activities

The aqueous extract of *B. frutescens* showed significant antisecretory activity with inhibitory rates of 86.0% at 300 mg/kg on cholera toxin-induced intestinal secretion in rat jejunal loops model [68]. *M. cordata* root powder could improve liver function in acute hepatic injuries. *M. cordata* could lessen the level of serum lactate dehydrogenase (LDH) and mortality rate of rats, while increasing the ratio of serum albumin/globulin (A/G), protecting cellular membrane effectively and inhibiting fibrosis in rats with chromic hepatic injury caused by tetrachloromethane. Furthermore, it enhanced the function of T and B lymphocytes [69]. The methanol extract of aerial parts of *M. cordata* exhibited strong antioxidative activity against total reactive oxygen species (ROS), with an IC_50_ value of 1.7 μg/mL, compared to the positive control Trolox (7.61 ± 0.12 μg/mL) [70]. The alkaloids from fruits of *M. cordata* collected in different regions possessed different antioxidant activities, with IC_50_ values ranging from 578 to 1,192 mg/L [71].

CHE and SAN, and their mixture (sanguiritrin) from *M. cordata* were inhibitors of aminopeptidase A and dipeptidyl peptidase IV. They inhibited amino peptidase N by 82%, 82%, and 88%, DPP IV by 38%, 62%, and 57%, respectively, at 50 mM. When bovine serum albumin (500 μg/mL) was added, the inhibition of both proteases by QBPA at 50 μM was significantly diminished, which suggested that strong interaction of CHE and SAN with bovine and human serum albumin was proved by electrophoretic determination of their respective conditional binding constants [72]. CHE, SAN and QBPA extract from *M. cordata* exerted differential inhibitory effects against hydrolytic activity of particular dipeptidyl peptidase (DPP)-like enzyme isolated from human blood plasma and from human and rat glioma cell lines. The low-MW form of DPP-IV-like enzyme activity, corresponding most probably with DPP-8, observed only in glioma cells but not in human plasma, was inhibited preferentially by CHE, SAN and the QBPA extract, indicating that some QBPA’s biological effects could be determined by tissue and cell type specific dipeptidyl peptidase IV-like molecules expression pattern [73]. SAN may, under appropriate conditions, increase the capacity of the enzymatic antioxidant defense system via activation of the p38 MAPK/Nrf2 pathway [64].

The alkaloids of *M. cordata* are the major active ingredients in Sangrovit, a phytogenic feed additive composed mainly of CHE and SAN. Jankowski *et al*. found that feeding broilers with 20 mg/kg of Sangrovit led to a significant increase of mucosal maltase, reduced duodenal villus height yet no change in pH in the small and lower intestine [74]. Despite lacking of improvement in final body weight, a low dose of dietary Sangrovit was found to exert positive effects on caecal metabolism of broilers [75]. In addition, macarpine (**31**), a QBPA described only in two species, was recently reported as promising fluorescent probe for labeling of cell nuclei at fluorescence microscopy and flow cytometry [31].

## 4. Toxicity and Side Effects

Modern pharmacological studies show that the QBPA bisulfates from *M. cordata* exhibited low toxicity in acute oral, acute inhalation and acute dermal toxicity tests, and no stimulating effect on the skin, but weak sensitization and severe irritation to the eyes [57]. It was reported in China that one person who ate bee slag containing *M. cordata* appeared to show symptoms like dizziness, tinnitus, numbness, and nausea [76]. There was another report that one female died after taking about 250 mL of fresh root decoction (125 g/250 mL) of *M. cordata*, supposedly used for curing joint pain [77].

The subchronic safety of sanguiritrin was assessed by feeding rats a diet containing 120 ppm (100 ppm QBPA) for 109 days, but no adverse effects were observed on rat organism, including no influence on the gut mucosal epithelium, liver tissue and any biochemical parameters. Oxidative stress did not manifest during the experiment [78]. The sensory evaluation of broiler breast and thigh meat did not reveal any negative influence of dietary supplementation with a 30 mg/kg dose of the alkaloid-containing preparation Sangrovitin for 5 week [79]. Rawling, *et al.* reported that low levels of Sangrovit (25–100 mg/kg) had a positive effect on tilapia growth performance with no apparent effects on carcass composition, hepatic function or health status [80]. Besides, the effects of daily administration of the extract from *M. cordata* (2 and 100 mg/kg feed, SAN:CHE = 3:1) in the diet on the health status of swine were evaluated, and the results showed that an average daily oral dose of alkaloids up to 5 mg/kg animal body weight proved to be safe [81]. The cytotoxicity analysis on primary cultures of human hepatocytes suggested that CHE and dihydrochelerythrine (**4**) were nontoxic up to 50 μM concentration [82]. However, the aqueous extracts of the dry roots of *M. microcarpa* showed toxic effect against growth of cultured algae (*P. subcapitata* and *S. quadricauda*) and cyanobacteria (*M. aeruginosa* and *S. leopoliensis*), with EC_50_ values of 626.90–984.81 mg/L [83].

SAN had been reported to form DNA adducts *in vitro* and to increase the levels of DNA single strand cleavage in the blood and bone marrow of mice injected intraperitoneally. There was no genotoxic effects of orally administrated 120 mg/kg feed Sangrovit in pigs or rats in a 90-day observation. *M. cordata* extract and/or Sangrovit induced no DNA damage to rat lymphocytes or hepatocytes after 90-days oral administration [84]. Some parameters of dextran sulfate sodium (DSS)-induced colitis were improved by adding 500 ppm Sangrovit to feed the rats. For example, it showed less severe damage to the colon mucosa and decreased histological colitis scores. What’s more, it showed a diminished expression of DSS-induced COX-2, significantly mitigating myeloperoxidase activity in colon tissue and reducing level of glutathione in erythrocytes [63]. Broadly, the Bocconieae alkaloids are considerable toxic to some extent, and for application of the extracts and pure alkaloids from the tribe attention must be paid to their side effects.

## 5. Discussion and Conclusions

A total of 75 alkaloids were described from the plants of the tribe Bocconieae so far, including benzophenanthridines, protoberberines, protopines, and others. Some exhibited such a variety of biological effects such as antitumor, insecticidal, and antimicrobial activities. SAN (**29**) has shown a promising future as a lead compound for the development of biological pesticides. Meanwhile, the side effects of the plants of this tribe should also be given adequate attention.

Currently, there are two opposing opinions when it comes to the classification system: one suggests that *Macleaya* and *Bocconia* should be classified as the tribe Bocconieae, which is documented in the Hutchinson System, while the other insists that *Macleaya* and *Bocconia* should be placed in Chelidonieae, because of the characteristic alkaloids, especially CHE and SAN, generally occurred in the Chelidonieae. Our present review reveals that there is no obvious difference in alkaloids observed in these two genera compared with those from Chelidonieae, and therefore provides chemotaxonomic evidence supporting the latter classification, although more substantial evidence is needed to determine the final taxonomy of these genera.

Since the plants of the tribe Bocconieae have ecological adaptability advantages, and are easy to cultivate and manage, their exploitation and utilization as natural antimicrobials have attracted much attention around the world. For instance, the alkaloids from *M. cordata* were used by United States as breath freshener and air freshener [85].

Overall, as an alkaloid-rich source, further in-depth research on the plants of the tribe Bocconieae, regarding their phytochemical investigation, determination of stereochemistry, *in vivo* and *in vitro* biological evaluation, and economic exploration, are of great significance and positively called for. 
